# Knowledge and practices towards rabies and determinants of dog rabies vaccination in households: a cross sectional study in an area with high dog bite incidents in Kakamega County, Kenya, 2013

**DOI:** 10.11604/pamj.2014.19.255.4745

**Published:** 2014-11-07

**Authors:** Gerald Mburu Mucheru, Gideon Mutie Kikuvi, Samuel Anyangu Amwayi

**Affiliations:** 1 Field Epidemiology and Lab Training Program (FELTP), Ministry of Health, Nairobi, Kenya; 2 College of Health Sciences, Jomo Kenyatta University of Agriculture and Technology, Nairobi, Kenya; 3 Kenya Ministry of Health, Nairobi, Kenya

**Keywords:** Rabies, dog, vaccination, household, survey, Knowledge, Practices, determinants, clusters, dog bite

## Abstract

**Introduction:**

An estimated 55,000 people die from rabies annually. Factors promoting dog vaccination, estimates of vaccination coverage and knowledge on rabies are important for effective rabies control. We sought to establish these estimates at household (HH) level and whether rabies knowledge is associated with proper control practices.

**Methods:**

Cross-sectional cluster survey with two-stage sampling was employed in Kakamega County to enroll HH members above 18 years. A set of questions related to rabies knowledge and practice were used to score participant response. Score above the sample mean was equated to adequate knowledge and proper practices respectively. Independent t-test was used to evaluate the differences of sample mean scores based on dog vaccination status. Bivariate analysis was used to associate knowledge to practices.

**Results:**

Three hundred and ninety HHs enrolled and had a population of 754 dogs with 35% (n = 119) HH having vaccinated dogs within past 12 months. Overall mean score for knowledge was 7.0 (±2.8) with range (0-11) and 6.3 (±1.2) for practice with range (0-8). There was a statistically significant difference in mean knowledge (DF = 288, p < 0.01) and practice (DF = 283, p = 0.001) of HH with vaccinated dogs compared to ones with unvaccinated dogs. Participants with adequate rabies knowledge were more likely to have proper health seeking practices 139 (80%) (OR = 3.0, 95% CI = 1.4-6.8) and proper handling practices of suspected rabid dog 327 (88%) (OR = 5.4, 95% CI = 2.7-10.6).

**Conclusion:**

Rabies vaccination below the 80% recommended for herd immunity. Mass vaccination campaign needed. More innovative ways of translating knowledge into proper rabies control practice are warranted.

## Introduction

Rabies is acute viral encephalitis affecting mainly carnivores and insectivorous bats but can affect any mammal. Case fatality rate is nearly always 100% once clinical signs appear. Rabies has a worldwide distribution in continental regions of Africa, Asia and the Americas but a few countries (e.g. Japan, Australia, New Zealand and The British Isles) are free of the disease due to successful eradication programs or their island status and enforcement of rigorous quarantine regulations [1]. In Africa, high rabies risk countries include Zambia, Angola, Namibia, Mozambique and Zimbabwe among others [2]. Globally, more than 15 million people receive rabies post exposure prophylaxis treatment [3] with an estimated that 55,000 people dying from rabies annually [4]. Africa and Asia record the highest human rabies deaths worldwide with an estimated 24,500 annual human deaths [5].

The first case of rabies in Kenya was diagnosed in 1912 and it was not until 1982 that the annual number of cases diagnosed rose to 200 [6]. The department of Livestock Development in Kenya has listed rabies as a notifiable disease with the zoonotic disease unit and the integrated disease surveillance and response strategy listing it as a priority disease. Determinant factors for dog vaccination, estimates of vaccination coverage and knowledge of rabies in households are useful elements for determining herd immunity and building effective control strategies. These estimates are lacking in most countries recording high dog bite and rabies cases including Kenya. In countries where domestic animals are not vaccinated against rabies, dogs are a source of 99% of human rabies deaths [7]. Adequate dog vaccination campaigns have been successful in causing dog rabies decline in high-density urban and rural areas of Kenya and Tanzania [8]. Despite numerous government and private rabies vaccination campaigns, rabies remains endemic in some parts of Kenya due to inadequate coverage and high dog turnover rates.

Dog registration and vaccination against rabies is a legal requirement and compulsory especially for dogs kept in urban areas under the revised rabies act chapter 365 [9]. Therefore, evaluation of vaccination coverage is vital to assessing adequacy of the vaccination programs. Studies on Vaccination coverage, dog ecology and models on dog vaccination done in Machakos District predicted that for rabies control to be effective, 59% of the dog population has to be vaccinated at any one time. For an annual vaccination cycle, at least 70% of the dog population needs to be vaccinated but for a bi-annual cycle, coverage of 60% would be adequate [10]. Therefore, with this herd immunity, the stray dog population ceases to be a major concern. Studies carried out in Eastern and Southern Africa has shown that for effective rabies control, dog ecological/demographic data are vital. These include the dog population density, dog population structure (age and sex) and the population characteristics of dogs which are mainly dog movements, restriction and dependency [10]. Apart from the occasional government vaccination clinics, no major steps have been taken by the government to promote awareness, proper dog handling and appropriate health seeking behavior in the event of dog bites in the community [11]. Lack of baseline data on knowledge, attitudes and practices regarding rabies could possibly be one of the reasons for delayed action. The objective of this study was to determine the rabies vaccination coverage among dogs at the household level, and establish whether the level of knowledge on rabies disease influences dog vaccination practices in Kakamega County of Kenya.

## Methods

**Study design** This was a cross-sectional cluster survey based on the World Health Organisation (WHO) Expanded Program on Immunization coverage.

### Study site

**Figure 1:**
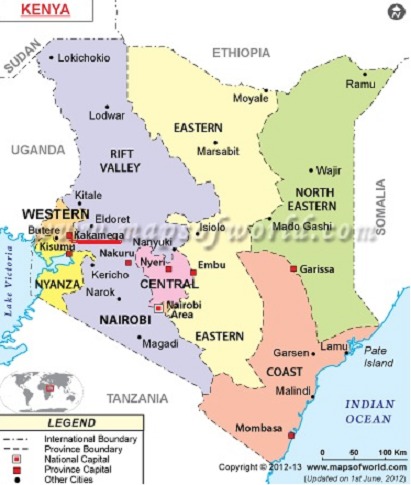
Location of Kakamega County, the study site, in Kenya

Figure 1 shows a map of Kakamega County which is located in the Western Part of Kenya. The county has a Total Population of 1,660,651 with 800,989 males and 859,662 females and has a total of 398,709 Households according to the two thousand and nine census report. It covers an area of 3,244.9 square kilometers. The Population density is 515 per square kilometer with 57% of the population is considered to live below the poverty line [11]. There are seven dog markets in the County namely: Nambacha, Lubao, Shikulu, Kakunga, Butali, Shinyalu and Matete. The markets are active all year round selling over 200 dogs per week with Lubao market selling the highest number of about 80 dogs per week. Each market has one market day in a week selling dogs in an open auction yard set aside for this purpose. There is normally a veterinary officer on site to offer vaccination services but the uptake by dog sellers is hardly 10%. The dog population distribution in the district is such that more dogs are concentrated around the municipality as a result of increased stray dog population with dog ownership further in the homesteads being evenly distributed [11].

### Sample size

A minimum sample size of 384 households calculated using the Cochran formula of 1977 [12]. The sample size required was estimated assuming an expected prevalence of 50% to achieve maximum sample size, a 95% CI and desired accuracy of 5% [12].

### Sampling scheme

A two-stage systematic random sampling method was done to recruit study participants. First stage: 30 clusters were selected from the master frame using simple random sampling where a cluster was the equivalent of an Enumeration Area (EA) with one Kenya National Bureau of Statistics (KNBS) Enumerator in-charge of one or more EA's. 2^nd^ stage: selection of households within clusters was done using systematic random sampling. The sampling interval (N/n) for each cluster was determined by dividing the total number of households (N) in each cluster by number of households to be interviewed for each cluster which was n= 13 since clusters had uneven number of households. At least 13 households and 7 dog owning households selected per cluster were sampled (requirement follows WHO EPI scheme to reduce effect of clustering in case some households owned ≥ 2 dogs). According to the 2009 Kenya National Housing and population census, the dog population in the County was not estimated.

### Data collection method

A household (eating from the same pot) member above 18 years was interviewed using a structured questionnaire. For purposes of assessing and scoring participants knowledge on rabies, six questions were used and covered a description of rabies, its mode of transmission and the outcome of disease, the range of species affected and how it can be either prevented or controlled. The highest mark achievable for knowledge was eleven and all respondents who got a score of seven and above were classified as being knowledgeable while those who scored below seven were classified as not knowledgeable. To assess whether the practices of participants reflected their knowledge of rabies, four questions were used covering length of time taken to present to hospital, practice towards suspect rabid animals and practice to carcass of suspect rabid animal. Participants were scored according to the completeness and accuracy of answers with correct answers scoring highest. Information on demographic characteristics of dog owners, determinants of dog vaccination practices such as dog restraint methods, time of vaccination campaign, frequency of vaccinating dog, accessibility of vaccination centers, dog vaccination status and other related dog demographic characteristics were also collected. Bivariate analysis was performed using the dog vaccination status as the outcome variable at 95% Confidence Interval and p < 0.05 as the level of significance. Data was analyzed using Epi-Info version 7 and Ms Excel.

## Results

A total of 390 households (HH) were enrolled during the study period and interviewed. Total HH interviewed from urban areas were194 (50%). Males interviewed during the study period were 205 (53%). The age range for all study participants interviewed during the study period was 18-99 years with a mean of 43 (±17) years. Most study participants(47%, n = 185) had attained upper primary education. A majority (61%, n = 237) of study participants interviewed were self-employed. The total dog owning households interviewed during the study period were338 (87%). Dog owning households that had a vaccinated dog regardless of the date of vaccination were 186 (55%). Out of these, 70 (38%) dogs had been vaccinated within the last six months and 49 (26%) had been vaccinated within the last twelve months. Out of all households with vaccinated dogs, only 119 (64%) households had dogs vaccinated within the last twelve months and out of all dog owning households, only 119 (35%) had dogs vaccinated within the last twelve months.

### Knowledge of rabies

**Figure 2:**
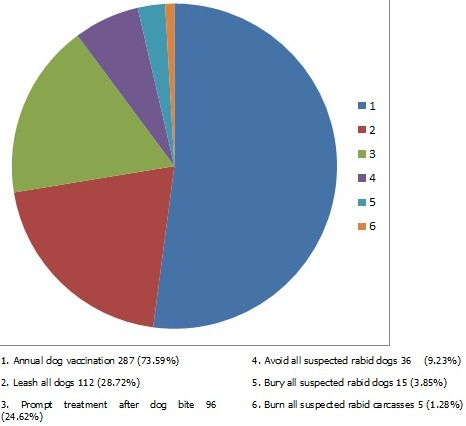
Shows the distribution of responses on rabies prevention methods preferred by respondents in Kakamega County, Kenya

Study participants who knew of rabies as a disease were 351 (90%) and out of these, 191 (49%) correctly described rabies as a disease while 127 (33%) described rabies as a change in the behavior of dog/animal. The source of information on rabies for 334 (86%) of the respondents was from family, friends, neighbors and colleagues. For animals that can be affected by rabies, 340 (87%) said dogs can be affected, 316 (81%) said man can get rabies. Only 114(29%) respondents said rabies can affect all animals. Respondents who thought animals can transmit rabies to humans were 317 (83%). Respondents who said rabies is transmitted through bites were 309 (79%) and those who responded rabies can be transmitted through scratches were 14 (4%). For all respondents interviewed, 138 (36%) said rabies is not curable. Most (89%, n = 348) respondents reported that exposure to rabies without treatment is fatal. Participants who responded to have seen a human rabies case were 158 (41%). Out of these, 156 (99%) reported that they saw the rabid person in real life. Most respondents mentioned dog vaccination and leashing as the preferred method of rabies prevention (Figure 2). For treatment of rabies/dog bites, 190 (49%) of respondents said anti-rabies is given, 84 (22%) said tetanus injection is given, 18 (5%) said antiseptics are applied to the bite wound while only 12 (3%) said the wound is washed with soap and water. Overall mean score for knowledge was 7.0 (±2.8) with range (0-11). Respondents who were knowledgeable on rabies were 261 (67%) while those who were unknowledgeable on rabies were 129 (33%).

### Practices on Rabies

For respondents who had ever been bitten by a dog (18%, n = 71) or who had a family member ever bitten by dog (30%. n = 116), only 125 (67%) went to hospital after the bite incident while 13 (7%) went to traditional healer. Respondents who claimed they would present to hospital immediately after a dog bite incident were 374 (96%) while 11 (3%) said they would present the following day. Out of all reasons given by all respondents as the reasons for dog bite victims not presenting to hospital, 266 (68%) said the cost of dog bite treatment is normally high, 22 (6%) said traditional healers are preferred while 11 (3%) said that the hospital could be far. In the case of a suspected rabid dog, 184 (47%) of the respondents reported that they would immediately kill the animal while 146 (37%) said they would report it to the veterinary officer. For those who reported that they would kill the suspected rabid dog, 175 (95%) said they would afterwards bury the carcass, 10 (5%) said they would throw away the carcass, 3/184 (2%) said they would burn the carcass while only 1 (0.54%) said they would cut the head and send it to the veterinary office.

Overall mean score for Practices on rabies was 6.3 (±1.2) with a range of (0-10). Respondents who had proper practice on the carcass of a suspected rabid dog were 177 (96%), respondents with proper first aid and medical care practice were 139 (80%), respondents with proper practice to the suspected rabid animal were 327 (88%) and those with proper practice of hospital presentation after dog bite were 387 (99%). Bivariate analysis was done to associate knowledge to practice with all the four practices as the exposure variables and knowledge of rabies as the outcome variable. Participants with adequate knowledge on rabies were more likely to have proper health seeking practices, 139 (80%) (OR = 3.0, 95% CI = 1.4-6.8) and proper handling practices of suspected rabid dog, 327 (88%) (OR = 5.4, 95% CI = 2.7-10.6) (Table 1). Independent t-test was used to evaluate the differences of sample mean scores based on dog vaccination status. There was a statistical significant difference in the mean knowledge(DF = 288, p < 0.01) and practice (DF = 283, p < 0.01) of HH with vaccinated dogs compared to ones with unvaccinated dogs.

**Table 1:** Relating knowledge of rabies to Proper practice among respondents, Kakamega County, Kenya, 2013

Variable	Knowledgeable	unknowledgeable	ODDS	95% CI	p-Value
**practice on rabid carcass**(yes)	95.07	97.67	0.45	0.05-3.8	0.75
(no)	4.93	2.33			
**medical attention/first aid practice** (yes)	84.96	65.00	3.0	1.36-6.8	0.01*
(no)	15.04	35.00			
**practice on suspect rabid dog** (yes)	94.19	75.00	5.4	2.7-10.6	0.000001*
(no)	5.81	25.00			
**practice of hospital presentation** (yes)	100.00	97.67	undefined	undefined	0.06
(no)	0.00	2.33			

### Determinant factors

Some of the factors that were significantly associated with vaccinated dogs in households on bivariate analysis are summarized in Table 2. On multivariate analysis, Factors that were independently and significantly associatedwith having a vaccinated dog at the household level are summarized in Table 3. They included having formal employment, knowledge of rabies as a disease including its fatal nature and knowledge of frequency of government sponsored vaccination clinics.

**Table 2:** Bivariate analysis of the vaccination determinant factors using the rabies vaccination statusas the outcome in households, Kakamega County, Kenya, 2013

VARIABLE	VACCINATED%	UNVACCINATED%	Odds ratio	95% CI	P value
**EMPLOYMENT STATUS**					
Formal employment (yes)	18.82	7.89	2.704	1.35-5.42	*0.006
(no)	81.18	92.11			
Dog bought (yes)	52.15	57.89	0.79	0.51-1.22	0.344
(no)	47.85	42.11			
Dog born in household(yes)	47.85	34.87	1.71	1.1-2.66	*0.021
(no)	52.15	65.13			
**DOG LEASHING**(yes)	79.46	72.19	1.50	0.9-2.46	0.15
(no)	20.54	27.81			
**DOG FEEDING**					
Food prepared specifically for dog (yes)	34.95	17.76	2.49	1.49-4.16	*0.0006
(no)	65.05	82.24			
Leftovers from house (yes)	50.54	44.74	1.26	0.82-1.94	0.34
(no)	49.46	55.26			
**NUMBER OF VACCINATION CLINICS**					
Twice (yes)	36.02	13.82	3.51	2.03-6.08	*0.000007
(no)	68.98	86.18			
Vet always available (yes)	23.12	3.29			
(no)	76.88	96.71	8.84	3.4-22.9	*0.000001
**AGE AT FIRST RABIES VACCINATION**					
<1 year and at 1 year (yes)	85.56	70.86	2.64	1.53-4.58	0.0006
(no)	13.44	29.14			
**DOG EVER BITTEN SOMEONE** (yes)	25.81	10.53	2.96	1.6-5.5	*0.0006
(no/don't know)	74.19	89.47			
**CHARGED FOR VICTIMS TREATMENT** (yes)	95.83	77.78	6.57	1.1-39.75	*0.07
(no)	4.17	22.22			
**KNOW RABIES DISEASE**(yes)	94.09	83.55	3.13	1.48-6.6	*0.003
(no)	5.91	16.45			
**OVERALL RABIES KNOWLEDGE**					
Knowledgeable (yes)	74.73	55.26	2.4	1.5-3.8	*0.00027
Unknowledgeable (no)	25.27	44.74			
**KNOW TRANSMISSION MODE**					
Through bites (yes)	84.95	69.08	2.53	1.49-4.29	*0.0007
(no)	15.05	30.92			
**PREVENTION OF RABIES**					
Know vaccinating will prevent (yes)	82.26	65.13	2.48	1.50-4.1	*0.0005
(no)	17.74	34.87			

**Table 3:** Summary of factors independently and significantly associated with having a vaccinated dog in households on Multivariate analysis, Kakamega County, Kenya, 2013

Having formal employment
Having food prepared specifically for dog in household
Knew of at least two annual vaccination clinics
Household had dog implicated to have bitten someone
Those who knew of a disease called rabies
Responded first rabies vaccination done when dog is <1/1yr old
Knew exposure to rabies without treatment leads to death
Would report a rabies case to the veterinary officers

## Discussion

The rabies vaccination coverage for this study was below the WHO recommended coverage of 80% required to achieve herd immunity. There was a statistically significant difference in the mean knowledge, and practice of Households with vaccinated dogs compared to ones with unvaccinated dogs with knowledgeable households being two and a half times more likely to have a vaccinated dog. Participants with adequate knowledge of rabies were also found to be more likely to have proper health seeking practices after dog bite incident and proper handling practices of suspected rabid dogs. Eighty six percent of the households were found to own one or more dogs. Implications: A high dog density combined with low vaccination coverage pauses a great public health risk in the event of a rabies outbreak.

Findings of a high dog density are different from those of a study of dog ecology and demography conducted in Machakos district in Kenya where 150 dog owning households were sampled and it was shown that dog ownership was common with 53-81% of households owning dogs [13] and different from findings of studies done in Ethiopia with 41% dog owning households [14], Thailand with 54% households owning dogs [12] and Tanzania with 22% dog ownership [15]. The difference could result from the existence of seven active dog markets in the study area resulting in more households being able to own a dog. The average number of dogs per household wasslightly higher than that of a study done in Thailand where average dogs per household were 0.9 and 1.7 dogs per dog owning household [12]. In this study, a higher percentage of dogs were bought probably due to the existence of a total of seven dog markets in the region. Respondents that owned a dog that was born in their household were twice more likely to have a vaccinated dog. Lengthy dog ownership could have led to better awareness of the benefits of regular dog vaccination.

The total percentage of Vaccinated dogs in the dog owning households interviewed was below the WHO recommended coverage for herd immunity and consistent with that of a study done in Ethiopia where 33% of dogs from dog owning households had been vaccinated [14] and another study done in Kenya in Machakos District [13]. More households reported having unmanageable dogs which could not be handled by family members compared to a study done in Machakos, Kenya, where 8% of the household dogs were unmanageable [13]. These dogs are likely to remain unvaccinated due to their ferocity. The findings on Households that reported leashing their dogs the whole day or letting them roam freely at some point during the day was consistent with a study done in Ethiopia where 48% were leashed at some point during the day with the roaming dog population different at 33% and different from another study done in Kenya where 69% of dogs from households interviewed were found to be roaming freely [13, 14]. A study done in Thailand by Kongkaew had 74% of owned household dogs roaming freely with 33% roaming freely in a community based study done in Sri Lanka [12, 16]. This roaming dog population has been shown to be the main cause of dog bite incidents leading to spread of rabies. Thirty seven percent of households fed their dog with the leftovers from their house with 21% preparing food specifically for the dog. These findings are different from those of a study done in Machakos, Kenya, where 95% of households fed their dogs on leftovers and only 5% of the households prepared food specifically for the dog [13], and in Thailand where 56% of households prepared food specifically for the dog [12] and in Ethiopia where 88% of the households fed their dogs on leftovers [14]. Households that prepared food specifically for the dog in this study were two and a half times more likely to have a vaccinated dog. Dog vaccination by these households could be an extension of humane dog treatment through proper dog food preparation and feeding.

Respondents who were aware that the government sponsored at least two annual vaccination clinics were three and a half times more likely to have a vaccinated dog in their household with respondents who reported that the veterinary personnel are always available to vaccinate their dogs anytime being nine times more likely to have a vaccinated dog. Household respondents who thought that the first rabies vaccination should be done when the dog is less than one year old were twice more likely to have a vaccinated dog. The result is different from that of a KAP survey done in Sri Lanka where only 36% responded first rabies vaccination be done when the dog is less than one year [17]. Households that reported their dog had at some point been implicated to have bitten someone were three times more likely to have a vaccinated dog probably due to awareness that post exposure prophylaxis is expensive after being charged for the victim's treatment. Households that knew of a disease called rabies were three times more likely to have a vaccinated dog due to awareness of its fatal nature if left untreated. this was consistent with studies in Sri Lanka (89%) and New Delhi (84%) on the fatal nature of rabies [17, 18]. The response on rabies knowledge is also consistent with the study by Sambo in Tanzania where 96% of respondents knew of rabies, the study by Kongkaew in Thailand where 93% of respondents knew of rabies and in Sri Lanka where 95% of respondents knew of rabies as a disease [12, 15, 17]. In this study, the respondents’ source of knowledge on rabies was from family, friends, neighbors and colleagues which was consistent with the study done in Tanzania where 70% of respondents reported neighbors, parents and friends as their main source of rabies knowledge [15]. Only 56% and 29% in Pakistan and Sri Lanka respectively reported friends and neighbors as their source [16, 17] with 36% of respondents in Thailand study reporting verbal propaganda as their source of knowledge on rabies [12]. Radio and TV had low response rates in this study compared to17% in Pakistan and 37% in Thailand [12, 16]. This shows that the media (Radio and TV) which is more accessible to a wider population has not been well utilized as a source of dispersing knowledge and awareness of rabies.

Respondents who had a secondary/tertiary education were two times more likely to have a vaccinated dog in the household which isconsistent with a study done in Tanzania [15] and Pakistan [16] where respondents with secondary education were associated with having better knowledge of rabies. This is probably due to awareness and access to information on rabies and the importance of dog vaccination. Respondents who were formally employed were three times more likely to have a vaccinated dog in the household. Having a stable income might be contributing to the ability to pay for vaccination services. In terms of dog acquisition and ownership, the results are different from a study on dog ecology and demography done in Machakos District in Kenya where 56% of the owned dogs were acquired as gifts given by friends or neighbors and only 9% had been bought [13]. In this study, owned dogs that had been adopted from the stray population were 9% which was similar to that of a study done in Thailand where 11% of the owned dogs had been adopted from the strays [12]. Respondents who thought rabies was mainly transmitted through animal bites were two and a half times more likely to have a vaccinated dog. This is probably due to the awareness that if their dog was unvaccinated and ended up biting someone, they would have to pay for the victims treatment. These findings are consistent with study in Ethiopia where 73% reported animal bites as main mode of rabies transmission with the study in Tanzania having 81% of these report [14, 15].

In terms of prevention, household respondents who thought annual vaccination of dogs, seeking medical attention immediately after dog bite incident, and that the treatment for dog bite was anti-rabies, were two times more likely to have a vaccinated dog. Knowledge of rabies prevention could stem from having experienced a dog bite incident either in the household or neighbors. The finding is consistent with one in Tanzania where 67% of respondents reported vaccination of dogs as the major mode of rabies prevention with 88% reporting the same in Sri Lanka. However more respondents (83%) in Tanzania reported they would seek medical attention immediately after dog bite incident [15]. For this study, awareness of the WHO recommended first aid measure of washing the bite wound with soap and water was the lowest compared to respondents from Sri Lanka 8%, respondents from the New Delhi community based study 32% and respondents from Thailand 70% [12, 17, 18]. The high response on these preferred first aid measure recommended by the WHO is possibly due to the fact that the studies were done in regions that had received some awareness campaigns prior to the studies.

Respondents who said that they would report a rabid dog to the veterinary officerswere two times more likely to have a vaccinated dog. This was different from the study in Tanzania where only 7% would report rabid case to livestock officer. In this study, there was no difference in the description of rabies between the urban and rural populations. This was consistent with the study in Tanzania which also found no difference in the description of rabies between urban and rural populations.

## Conclusion

Rabies vaccination is below the recommended 80% for achievement of herd immunity. Household respondents who knew of two annual government vaccination clinics and those who said the veterinary officers are always available for vaccination were more likely to have a vaccinated dog. The veterinary department needs to sensitize the community on frequency, location and availability of veterinary vaccination centers to increase the rabies vaccination coverage, which currently stands at 35%, to at least 70%. Alternatively, the department could increase the number of veterinary officers to be present consistently across the county as those who reported that officers were always available were significantly associated with having a vaccinated dog.

Participants with adequate knowledge on rabies were more likely to have proper health seeking practices and proper handling practices of suspected rabid dog. Awareness campaigns on rabies etiology, range of clinical symptoms in man and animals, critical first aid measures of washing any bite wound with copious amounts of soap and water, treatment and control measures are warranted. The government should also aim to sensitize the community to report any rabid or suspected rabid animals to the veterinary personnel. There is need for education of dog owning households on responsible dog ownership such as proper dog feeding and leashing to prevent roaming and scavenging, enforcing animal control by-laws on dog restrain/leashing in collaboration with the county authorities and regular and timely rabies vaccination of all dogs more than three months old.
